# A comparative study of the incidence of in-hospital cardiopulmonary resuscitation on Monday–Wednesday and Thursday–Sunday: Retrospective analysis in a tertiary care hospital

**DOI:** 10.1097/MD.0000000000009741

**Published:** 2018-02-09

**Authors:** Tak Kyu Oh, Young Mi Park, Sang-Hwan Do, Jung-Won Hwang, You Hwan Jo, Jin Hee Kim, Young-Tae Jeon, In-Ae Song

**Affiliations:** a Interdepartment of Critical Care Medicine, Seoul National University Bundang Hospital; b Department of Anesthesiology and Pain Medicine, Seoul National University Bundang Hospital; c Medical Research Collaborating Center, Seoul National University Bundang Hospital; d Department of Emergency Medicine, Seoul National University Bundang Hospital, Seongnam, Korea.

**Keywords:** cardiopulmonary resuscitation, critical care, hospitals, resuscitation

## Abstract

Because most medical staff work from Monday–Friday, it is possible that they are relatively more fatigued and less capable of providing emergency supportive services on Thursday–Sunday (Thu–Sun) than on Monday–Wednesday (Mon–Wed). In this study, we aimed to analyze the incidence of in-hospital cardiopulmonary resuscitation (CPR) to determine if it differed between Thu–Sun and Mon–Wed.

This retrospective observational study of in-hospital CPR was performed during 2012 to 2016 among inpatients at the Seoul National University Bundang Hospital. The primary outcome was the incidence of in-hospital CPR per 1000 inpatients in the Mon–Wed and Thu–Sun periods. Secondary outcomes included differences in the CPR incidence by time of day and season.

In the study, 1195 cases of in-hospital CPR were included. The incidence of in-hospital CPR per 1000 inpatients was significantly higher on Thu–Sun (mean: 0.595, 95% confidence interval [CI]: 0.564–0.626) than on Mon–Wed (mean: 0.505, 95% CI: 0.474–0.536, *P* < .001). There were no seasonal variations in the incidence of in-hospital CPR. However, in-hospital CPR was most frequently performed between 16:00 and 24:00, and the return of spontaneous circulation (ROSC) rate was the lowest among cases that occurred between 0:00 and 8:00. In addition, the ROSC rate was lowest among female patients, patients with cardiac arrest, and after in-hospital CPR performed on a Sunday.

The incidence of in-hospital CPR per 1000 inpatients was significantly higher on Thu–Sun than on Mon–Wed. No seasonal variations were observed in the incidence of in-hospital CPR, but the data suggest circadian variations and differences in ROSC rates.

## Abstract 2

Supplemental Digital Content is available in the text

## 1 Introduction

Patients who require cardiopulmonary resuscitation (CPR) within the hospital generally differ from those who require out-of-hospital CPR, and in-hospital CPR is known to be an important determinant of in-hospital mortality.^[1]^ Epidemiologic studies have reported that the incidence of in-hospital CPR among elderly individuals is about 2.73 per 1000 inpatients, and have suggested that it is difficult to improve the survival rate of in-hospital CPR.^[2,3]^ Unlike cases requiring out-of-hospital CPR, in-hospital CPR may be preventable in some cases and its incidence is closely related to medical staff. Furthermore, although out-of-hospital CPR is influenced by a circadian rhythm or weekday effect,^[4,5]^ no data are currently available regarding circadian variations in or a weekday effect on in-hospital CPR. Therefore, there is an urgent need for studies investigating the circadian variations of in-hospital CPR.^[6]^

Most medical staff work from Monday–Friday; medical care on the weekends is commonly provided by on-duty medical staff. Medical staff fatigue has been closely associated with poor patient outcomes.^[7]^ Previous studies have also shown that patient mortality increases on weekends (Saturday–Sunday), which are associated with lower number of on-duty staff and a higher proportion of trainees working as primary physicians.^[8,9]^ Therefore, we hypothesized that in-hospital CPR may occur more frequently on Thu–Sun, when the medical staff are more fatigued and the number and quality of on-duty physicians may be lower, than on Mon–Wed.

The main aim of this study was to examine the differences in in-hospital CPR between Thu–Sun and Mon–Wed. In addition, we aimed to investigate daily, monthly, and seasonal circadian variations in the incidence of in-hospital cardiac arrest. We hypothesized that the incidence of in-hospital CPR would be higher on Thu–Sun than on Mon–Wed and that there would be daily and seasonal variations in the incidence.

## 2 Methods

This was a retrospective cohort study of adult and pediatric inpatients at the Seoul National University Bundang Hospital (SNUBH) in Korea who received in-hospital CPR from 2012 to 2016. Patients were excluded if they had an incomplete CPR-related medical record or if they underwent CPR in the emergency room (ER). The SNUBH is a 1266-bed tertiary care hospital; as of March 2017, there were 1164 beds in the ward and 102 beds in the intensive care unit (ICU). It is a large teaching center located in Seongnam, Gyeongi-do (https://www.snubh.org/dh/en). Since 2003, it has been managing its medical records via an electronic medical record system and has been recording the accurate time and outcome of CPR using a CPR report. The CPR report is an SNUBH record that provides detailed information regarding the time, location, performer, precipitating event, and outcome of CPR.

The electronic medical record system was used to collect data on the year, date, day of the week, and time of day at which the in-hospital CPR occurred, in addition to the patient's height (cm) and weight (kg), arrest type (cardiac or noncardiac), return of spontaneous circulation (ROSC), total length of hospital stay, and length of ICU stay. The monthly count of inpatients and average daily number of inpatients were the average numbers of inpatients for the month or day, respectively. The number of inpatients was calculated using the following formula: total number of patients admitted in the ward or ICU before the study start date (no plans for discharge in the study date range) + new admission in the study date range − discharge in the study date range. Data were collected systematically by the SNUBH medical information team consisting of professional medical record technicians. The data collectors were blinded to the primary outcome during data collection.

The primary outcome investigated during this study was the incidence of in-hospital CPR per 1000 inpatients. We stratified the data by day of occurrence (Thu–Sun vs. Mon–Wed, Fri–Sun vs. Mon–Thu, and Sat–Sun vs. Mon–Fri) to detect any differences in CPR incidence. The secondary outcome was the seasonal distribution of in-hospital CPR per 1000 inpatients, daily distribution of all CPR cases, and factors related to ROSC. Seasons were defined as follows: spring (March–May), summer (June–August), fall (September–November), and winter (December–February). The time of day was divided into three 8-h intervals: nighttime (0:00–08:00), office hours (08:00–16:00), and evening time (16:00–24:00).

### 2.1 Statistical analysis

To compare the difference in hospital CPR incidence, the number of hospitalized patients according to each day and season was taken into account to offset the patient bias at specific times. Accordingly, we evaluated the incidence of in-hospital CPR per 1000 inpatients. We concluded that autocorrelation characteristics were not affected by the previous occurrence of CPR. Therefore, we did not require seasonal correction method or any special technique to analyze the time-series data.

A comparison of the frequency of in-hospital CPR per 1000 inpatients in the first half of the week (Monday–Tuesday–Wednesday) and later (Thursday–Friday–Saturday–Sunday) was performed by the 2-proportion Z-test. The frequency of occurrence in the 4 seasons was also evaluated in the same way.

Univariate logistic regression analysis was used to determine whether there were significant death events among CPR patients during a day. For this, a day was divided into 3 intervals as night time (0:00–08:00), office hour (08:00–16:00), and evening time (16:00–24:00), which were used as categorical independent variables. Finally, factors associated with death after CPR were estimated using univariate logistic regression and multivariate logistic regression. First, all the predictor variables were ranked according to their *P* value obtained from the univariate logistic regression analysis. Using this ranked data, we found that sex, age, weight, time of occurrence categorized into 3 intervals, day of occurrence, place of occurrence, and reason of occurrence could predict the event of death. As the number of observations of total data exceeded 1000, 7 variables considered to be significant in the univariate analysis were considered in the multivariate logistic regression.

*P* values were used to determine the statistical significance; *P* value ≤0.05 was considered significant. All statistical analyses were performed using open-source statistical software R, version 3.3.2 (http://www.R-project.org) with R packages.

This study was approved by the Institutional Review Board of SNUBH (IRB No. B-1703/388-107). The need for informed consent was waived because of the retrospective study design.

## 3 Results

There were 1228 cases of in-hospital CPR between 2012 and 2016. Eight cases were excluded as they had incomplete medical records and 25 cases were excluded because CPR was performed in the ER; therefore, a total of 1195 cases of in-hospital CPR were included in the study. The monthly count of inpatients and average daily number of inpatients used to calculate the incidence of in-hospital CPR per 1000 inpatients between 2012 and 2016 are presented in Appendix 1.

**Figure 1:**
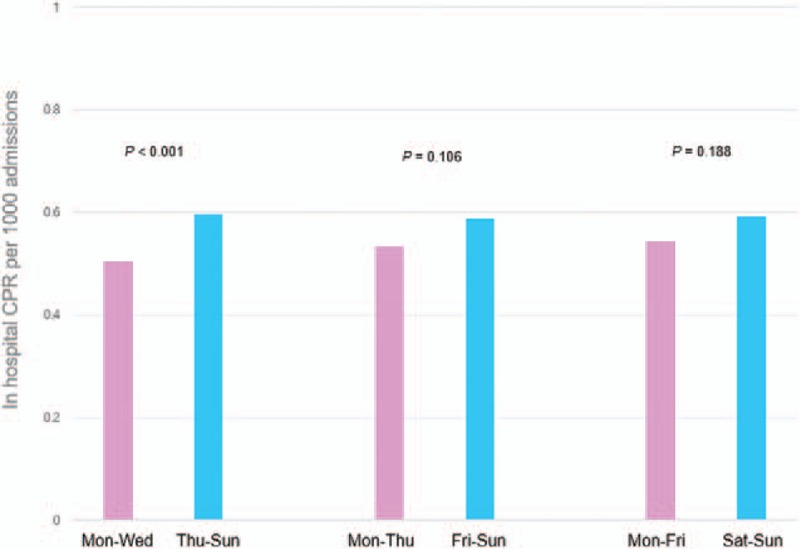
In-hospital CPR per 1000 admissions between Thu–Sun and Mon–Wed. Thu–Sun: Thursday, Friday, Saturday, and Sunday. Mon–Wed: Monday, Tuesday, and Wednesday. Fri–Sun: Friday, Saturday, and Sunday. Mon–Thu: Monday, Tuesday, Wednesday, and Thursday. Sat–Sun: Saturday and Sunday. Mon–Fri: Monday, Tuesday, Wednesday, Thursday, and Friday. The bars represent in-hospital CPR per 1000 inpatients. Two-proportion Z-test was used for comparing the frequency of in-hospital CPR per 1000 inpatients. CPR = cardiopulmonary resuscitation.

In-hospital CPR was frequently performed on female patients, in wards, and on patients with cardiac arrest, and ROSC was achieved in 72.3% of in-hospital CPR cases (Table 1). The likelihood of in-hospital CPR was significantly higher on Thu–Sun than on Mon–Wed (mean number of CPR cases per 1000 inpatients: 0.595, 95% confidence interval [CI]: 0.564–0.626 vs. mean number of CPR cases per 1000 inpatients: 0.505, 95% CI: 0.474–0.536, respectively; *P* < .001; Fig. 1). However, there was no significant difference in the mean number of CPR cases per 1000 inpatients between Mon–Thu and Fri–Sun (*P* = .106) or between Mon–Fri and Sat–Sun (*P* = .188). Appendix 2 shows distribution of in-hospital CPR cases per 1000 admissions according to days of the week (mean: 0.557 ± 0.057, Thursday: 0.62, Friday: 0.58, Saturday: 0.53, Sunday: 0.65, Monday: 0.53, Tuesday: 0.51, and Wednesday: 0.48).

**Table 1:**
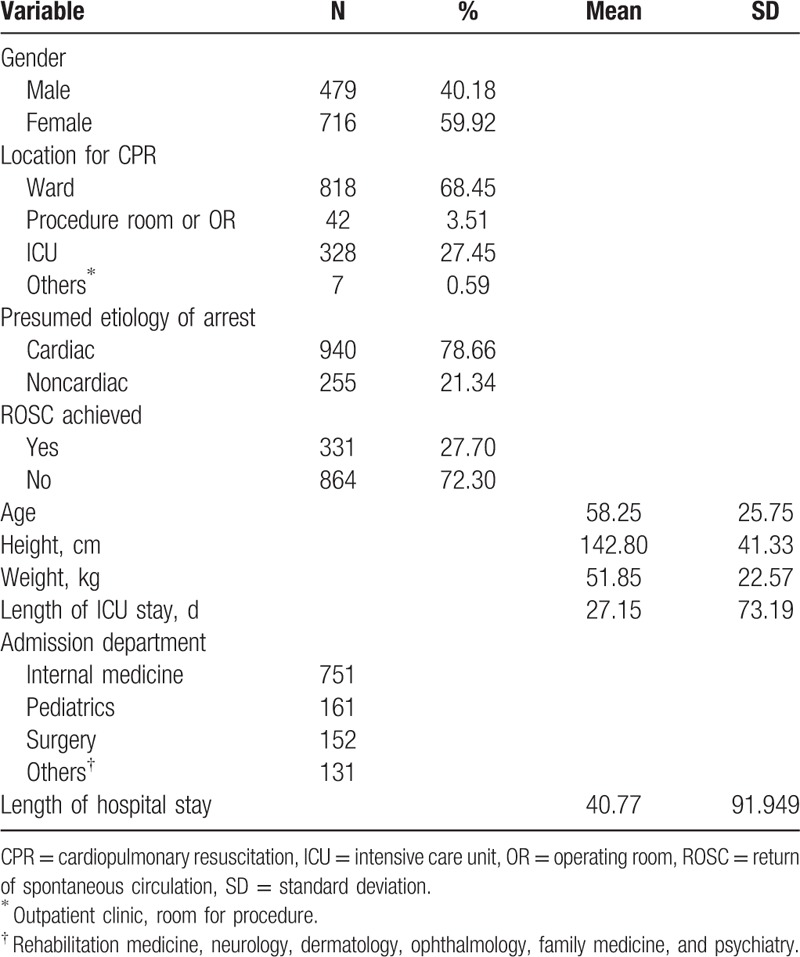
The characteristics of the in-hospital CPR cases.

**Figure 2:**
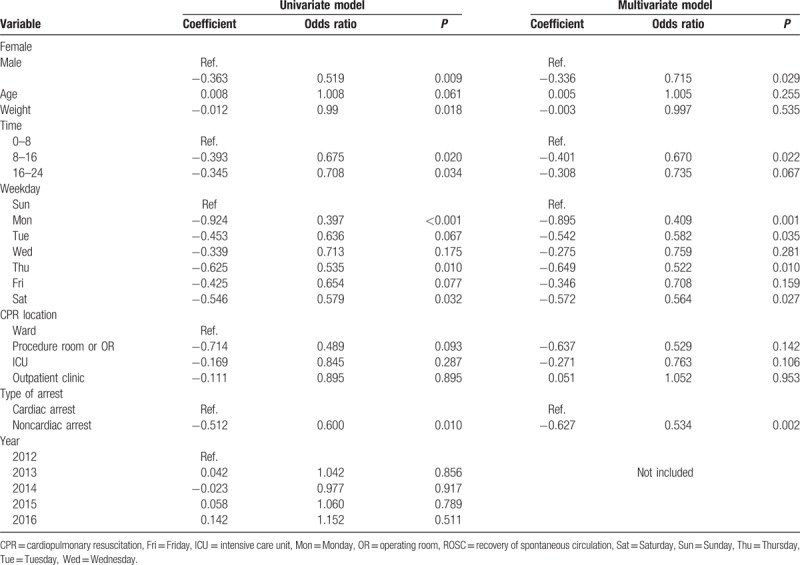
Diurnal variation in in-hospital CPR and ROSC rate. The bars represent the numbers of in-hospital CPR cases according to 8-h intervals in a day (night-time [0:00–08:00], office hours [08:00–16:00], and evening-time [16:00–24:00]). Patient mortality (failure to achieve ROSC) was the highest between 0:00 and 8:00 (126/386, 33%), followed by 16:00 to 24:00 (114/425, 27%) and 8:00 to 16:00 (98/384, 26%). Univariate logistic regression and multivariate logistic regression analyses were used to compare the probability of failure to achieve ROSC according to the daily 8-h intervals. The probability of failure to achieve ROSC was lower among those who underwent CPR between 8:00 and 16:00 (∗, 33%) and between 16:00 and 24:00 (∗∗, 26%) than in those who underwent CPR between 0:00 and 8:00 (∗*P* = .022 and ∗∗*P* = .067). CPR = cardiopulmonary resuscitation, ROSC = return of spontaneous circulation.

**Figure 3:**
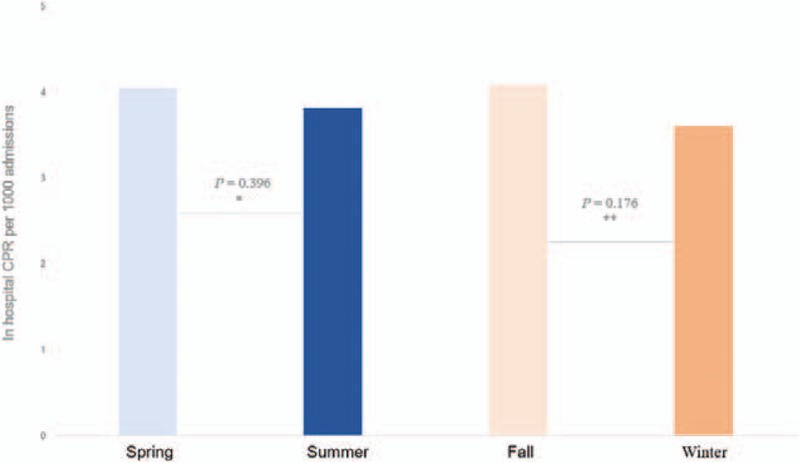
Seasonal variations in the incidence of in-hospital CPR per 1000 admissions. The bars represent in-hospital CPR cases per 1000 admissions according to 4 seasons. The horizontal blue line represents average number of in-hospital CPR cases per 1000 admissions per season. Two-proportion Z-test was used for comparing the frequencies of in-hospital CPR per 1000 inpatients. Spring versus summer: 4.05 versus 3.82, ∗*P* = .325, and fall versus winter: 4.09 versus 3.60, ∗∗*P* = .152. CPR = cardiopulmonary resuscitation.

In-hospital CPR was most frequently performed between 16:00 and 24:00 and least frequently performed between 0:00 and 8:00. However, patient mortality (failure to achieve ROSC) was the highest between 0:00 and 8:00 (126/387, 33%), followed by 16:00 to 24:00 (114/434, 26%) and 8:00 to 16:00 (99/399, 25%) (Fig. 2). On average, 3.97 ± 0.23 cases of CPR were performed each season, with no significant seasonal variations in the incidence of CPR (spring vs. summer, *P* = .396; fall vs. winter, *P* = .176; Fig. 3).

The probability of failure to achieve ROSC was approximately 30% lower in male patients than in female patients (*P* = .03), and the probability of mortality was lower among those with CPR performed between 8:00 and 16:00 (33%) and between 16:00 and 24:00 (27%) than those with CPR performed between 0:00 and 8:00 (*P* = .024 and *P* = .063, respectively; Table 2). In addition, the probability of failure to achieve ROSC after CPR significantly decreased over the course of a week beginning with Sunday (Monday 60%, Tuesday 42%, Thursday 48%, Saturday 43%; *P* = .001, *P* = .035, *P* = .010, *P* = .029, respectively). The probability of failure to achieve ROSC was 90% lower when CPR was performed in the ER than when it was performed in a ward (*P* = .022), and was 47% lower for patients with noncardiac causes versus those with cardiac arrest (*P* = .002). There were no differences in ROSC rates for season and year of in-hospital CPR (*P* > .05).

**Table 2:**
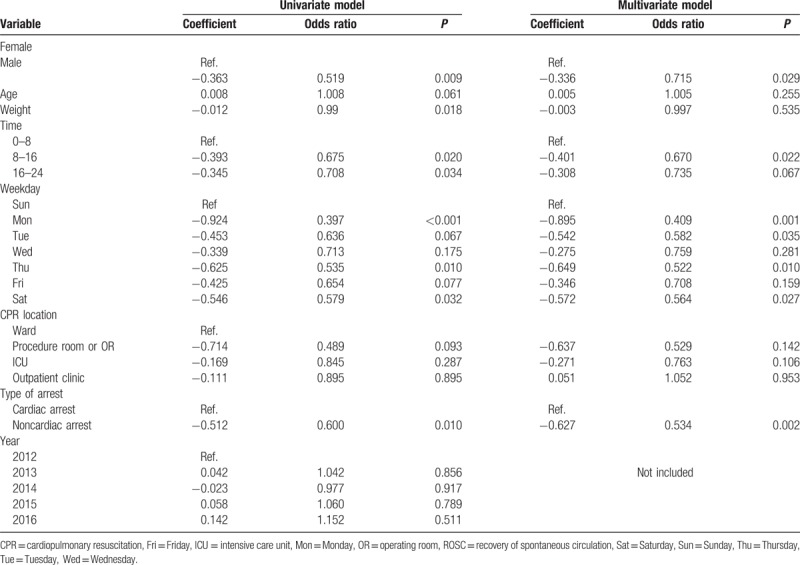
Univariate and multivariate analyses for failure in ROSC.

## 4 Discussion

This study confirmed that the incidence of in-hospital CPR per 1000 inpatients is higher on Thu–Sun than on Mon–Wed. This is the first study in which fatigue among medical staff was considered when investigating the weekend effect. Our results are significant as they were obtained using data from a 1266-bed tertiary care teaching hospital. In-hospital CPR was most frequently performed in the evening, while the ROSC failure rate was the highest at night. The incidence of CPR per 1000 inpatients did not vary over seasons. These results are in contradiction to previous findings that suggested the presence of seasonal variation in out-of-hospital CPR.^[10,11]^ We found that patient sex, day of week, nighttime, and place of CPR were significantly associated with ROSC.

The higher incidence of in-hospital CPR per 1000 inpatients on Thu–Sun compared with Mon–Wed could be due to the higher levels of fatigue experienced by medical staff on Thu–Sun compared with that on Mon–Wed. It is difficult to substantiate a direct relationship between fatigue/tension and incidence of in-hospital CPR, and thus, only a few studies have investigated this. It is hypothesized that higher fatigue and lower support for patients among the medical staff on Thursdays and Fridays increases the rate of in-hospital CPR during those 2 days. A Canadian epidemiologic study reported that the outcomes of ambulatory surgery were worse on Fridays than on Mondays.^[12]^ In general, the condition of patients at risk of in-hospital CPR is more severe than that of patients undergoing ambulatory surgery, indicating that they require special attention and high levels of support from the trained medical staff.^[3]^ Thus, the rate of in-hospital CPR on Thursdays and Fridays is likely to be affected by factors related to the medical staff rather than the patients.

The impact of the weekend effect on the rate of in-hospital CPR remains controversial. Although the higher rates of in-hospital CPR on weekends may be caused by the lower number of staff and higher proportion of on-duty trainees,^[8]^ some reports have suggested that the condition of patients admitted on the weekends is more severe compared with the condition of those hospitalized during weekdays.^[13,14]^ Since the in-hospital CPR rate was quite high and the ROSC rate was the lowest on Sundays, the effect of the severity of patients’ conditions cannot be ruled out.^[9]^ Moreover, since October 2012, SNUBH has established a rapid response system to prevent sudden cardiopulmonary arrest; however, this system is not active on Sundays.^[15]^ Considering that the rapid response system prevents a considerable number of in-hospital CPR cases, the high incidence of in-hospital CPR and the low ROSC rates on Sundays can be attributed to the medical staff.^[15]^ Therefore, the spike on in-hospital CPR on Sunday might be explained by the nonavailability of the part-time rapid response system on Sunday.

However, our study shows another in-hospital CPR spike on Thursday, despite the availability of the part-time rapid response team. Moreover, a significant difference in in-hospital CPR was found for Mon–Wed versus Thu–Sun but not for Mon–Thu versus Fri–Sun or Mon–Fri versus Sat–Sun. This indicates that the incidence of CPR in our hospital was highest on Thursday. None of the previous studies reported adverse outcomes associated with Thursday. Therefore, fatigue alone cannot explain this “Thursday effect.” One potential explanation for this effect is that the medical staff at SNUBH intentionally attempted to hospitalize fewer patients with serious conditions on Friday, knowing that previous studies had indicated the existence of an adverse “weekend effect”^[13,14]^ or on the basis of their own experiences. However, further research is needed to confirm this hypothesis.

We also investigated the daily circadian and seasonal effects on in-hospital CPR. We found that CPR was most frequently performed in the evening between 16:00 and 24:00 and the ROSC rate was the lowest in early morning between 0:00 and 8:00. Our findings are similar to those from previous studies, which showed that patients were more likely to require CPR and were more likely to experience the poorest outcomes during the night.^[16,17]^ In addition, the ROSC rate of CPR did not vary across seasons or years but did vary by time of day. A previous study^[17]^ showed that the ROSC rates were lower during the night than during the day for both cardiac and respiratory arrest. Furthermore, ROSC rates were lower for CPR performed on Sundays and for female patients. The lower ROSC rate for female patients could potentially be explained by an age-based hypothesis. In general, females have a much longer life expectancy than males do in South Korea, and in our study, the average age of patients who underwent CPR in the study period was 55.23 ± 21.25 for male patients and 62 ± 24.38 for female patients. However, further research is needed to test this hypothesis.

Our study has a few limitations. First, the retrospective design leaves the study susceptible to selection bias. Second, we could not substantiate the assumption that the medical staff are more fatigued and less supportive on Thu–Fri and that the patients admitted on Sat–Sun have more severe conditions. Third, we could not obtain objective data on the hospitals’ medical personnel management during the study period. Fourth, we could not establish clear etiologies for the occurrences of arrest (e.g., sepsis, embolism). Fifth, the reason for the spike in in-hospital CPR on Thursday could not be established. Finally, CPR was performed by medical staff from various departments and not by a single CPR team, and we did not evaluate the data according to the team performing the CPR. Nevertheless, this study is meaningful as it is the first study reflecting the effects of Thu–Fri, in addition to the weekend effect, on the rate of in-hospital CPR. Moreover, this study systematically reported the effects of season and time of day on the rate of in-hospital CPR in a tertiary care teaching hospital over a 5-year period.

In conclusion, the incidence of in-hospital CPR per 1000 inpatients was higher on Thu–Sun than on Mon–Wed. Furthermore, the data analysis results suggested that the incidence of in-hospital CPR did not vary seasonally but did vary by time of day; we also observed differences in ROSC rates. Additional studies are needed to investigate the impact of day of the week on in-hospital CPR.

## Acknowledgement

We would like to express our sincere appreciation to the members of the “rapid response team, intensivists, CPR team, and the Emergency department at Seoul National University Bundang Hospital.

## Supplementary Material

Supplemental Digital Content

## Supplementary Material

Supplemental Digital Content
