# Supplementary material for: A comparative study of the incidence of in-hospital cardiopulmonary resuscitation on Monday–Wednesday and Thursday–Sunday: Retrospective analysis in a tertiary care hospital
Source: Medicine (Baltimore). 2018 Feb 9;97(6):e9741. doi: 10.1097/MD.0000000000009741 (PMC5944682; doi:10.1097/MD.0000000000009741)
Supplement: Supplemental Digital Content [file medi-97-e9741-s001.docx]

Appendix 1. Admissions from 2012 to 2016 in SNUBH (per month and per weekday)

|  | January | | Februrary | March | | April | May | | June | July | | August | September | | October | November | | December |
| --- | --- | --- | --- | --- | --- | --- | --- | --- | --- | --- | --- | --- | --- | --- | --- | --- | --- | --- |
| 2012 | 4432 | | 4090 | 4166 | | 3968 | 4211 | | 3997 | 4379 | | 4404 | 3950 | | 3941 | 4085 | | 4129 |
| 2013 | 4576 | | 4038 | 4467 | | 4441 | 4531 | | 4512 | 4969 | | 4779 | 4392 | | 4663 | 4634 | | 5032 |
| 2014 | 5103 | | 4836 | 4874 | | 5032 | 4909 | | 4930 | 5656 | | 5334 | 4798 | | 5180 | 5021 | | 5831 |
| 2015 | 6118 | | 5113 | 6142 | | 5816 | 5328 | | 4872 | 5768 | | 6169 | 5460 | | 5730 | 5755 | | 6175 |
| 2016 | 6484 | | 5681 | 6393 | | 5849 | 6148 | | 5900 | 6101 | | 6271 | 5556 | | 5914 | 6045 | | 6312 |
| Monday | | Tuesday | | | Wednesday | | | Thursday | | | Friday | | | Saturday | | | Sunday | |
| 1192.77 | | 1243.71 | | | 1258.18 | | | 1242.85 | | | 1205.11 | | | 1069.5 | | | 1041.96 | |
