# Supplementary figures and images for: A comparative study of the incidence of in-hospital cardiopulmonary resuscitation on Monday–Wednesday and Thursday–Sunday: Retrospective analysis in a tertiary care hospital
Source: Medicine (Baltimore). 2018 Feb 9;97(6):e9741. doi: 10.1097/MD.0000000000009741 (PMC5944682; doi:10.1097/MD.0000000000009741)

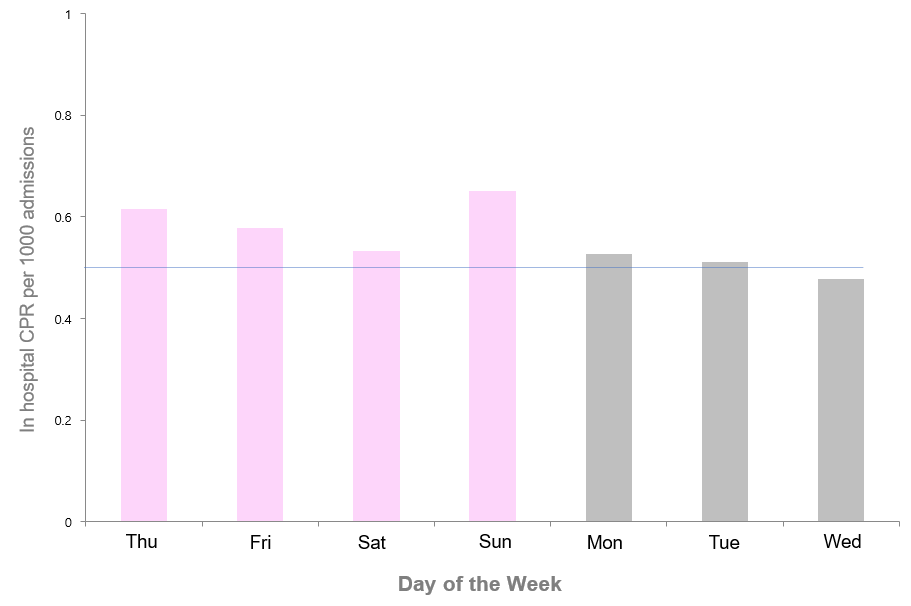

Supplement: Supplemental Digital Content [file medi-97-e9741-s002.tif]
